# INTEGRATING EMERITI PROFESSORS INTO AN AGE-INCLUSIVE LEARNING ACTIVITY FOR UNDERGRADUATE STUDENTS

**DOI:** 10.1093/geroni/igae098.0989

**Published:** 2024-12-31

**Authors:** Ramraj Gautam, karen Devereaux Melillo, Lisa Abdallah

**Affiliations:** University of Massachusetts Lowell, Lowell, Massachusetts, United States; University of Massachusetts Lowell, Lowell, Massachusetts, United States; University of Massachusetts Lowell, Lowell, Massachusetts, United States

## Abstract

Engaging retired faculty is at the forefront of the Age-Friendly University (AFU) principles and the community domain of the Age-Friendly Inventory and Campus Climate Survey (ICCS) (Silverstein, et al., 2022). Engaging Retired/Emeriti Professors (REP) at UMass Lowell (UML) began in 2020 when the ICCS survey identified “involving retired faculty in university activities” (AFU Principle 9) as an area for improvement. In turn, we surveyed the UML REP in 2021. In 2022, a sequential mixed method study overwhelmingly showed REP’s interest in intergenerational learning activities (IGL), supporting the AFU principle 4 (promote IGL between learners of all ages). An IGL activity was designed in the undergraduate Introduction to Gerontology course, where a team of five students conducted two one-hour online interviews with a REP. This presentation reports findings of students’ open-ended responses regarding their experience and the impact this IGL activity had on their attitudes/perceptions of aging. This University IRB-approved study sample included 94 students. Qualitative content analysis coding (Vaismoradi et al., (2016) was performed by three authors, to reach a consensus on individually identified themes. The findings revealed two major themes: Positive attitudes and perceptions towards aging; and a greater understanding of life in retirement. Likewise, the open-ended responses revealed most students (75%) expressed having a positive attitude and perception toward older adults while about 25% expressed either neutral or limited impact. These findings will be discussed in relation to the valuable role REP can play in intergenerational learning activities within the University while fostering a more age-inclusive environment.

